# Study protocol: combined N-of-1 trials to assess open-label placebo treatment for antidepressant discontinuation symptoms [FAB-study]

**DOI:** 10.1186/s12888-023-05184-y

**Published:** 2023-10-13

**Authors:** Amke Müller, Stefan Konigorski, Carina Meißner, Tahmine Fadai, Claire V. Warren, Irina Falkenberg, Tilo Kircher, Yvonne Nestoriuc

**Affiliations:** 1 Clinical Psychology, Helmut-Schmidt-University/University of the Federal Armed Forces Hamburg, Holstenhofweg 85, 22043 Hamburg, Germany; 2 Institute of Systems Neuroscience, University Medical Center Hamburg-Eppendorf, Martinistraße 52, 20246 Hamburg, Germany; 3 Digital Health – Machine Learning Group, Hasso-Plattner-Institute for Digital Engineering, Potsdam, Germany; 4 Hasso Plattner Institute for Digital Health at Mount Sinai, Icahn School of Medicine at Mount Sinai, New York, NY 10029 USA; 5 Department of Statistics, Harvard University, 150 Western Ave, Boston, MA 02134 USA; 6 Department of Psychiatry and Psychotherapy, University of Marburg, Marburg, Germany

**Keywords:** Antidepressant withdrawal, SSRI, SNRI, Side-effects, Depressive disorder, N-of-1, Placebo, Nocebo, Treatment expectation

## Abstract

**Background:**

Antidepressant discontinuation is associated with a broad range of adverse effects. Debilitating discontinuation symptoms can impede the discontinuation process and contribute to unnecessary long-term use of antidepressants. Antidepressant trials reveal large placebo effects, indicating a potential use of open-label placebo (OLP) treatment to facilitate the discontinuation process. We aim to determine the effect of OLP treatment in reducing antidepressant discontinuation symptoms using a series of N-of-1 trials.

**Methods:**

A series of randomized, single-blinded N-of-1 trials will be conducted in 20 patients with fully remitted DSM-V major depressive disorder, experiencing moderate to severe discontinuation symptoms following antidepressant discontinuation. Each N-of-1 trial consists of two cycles, each comprising two-week alternating periods of OLP treatment and of no treatment in a random order, for a total of eight weeks. Our primary outcome will be self-reported discontinuation symptoms rated twice daily via the smartphone application ‘StudyU’. Secondary outcomes include expectations about discontinuation symptoms and (depressed) mood. Statistical analyses will be based on a Bayesian multi-level random effects model, reporting posterior estimates of the overall and individual treatment effects.

**Discussion:**

Results of this trial will provide insight into the clinical application of OLP in treating antidepressant discontinuation symptoms, potentially offering a new cost-effective therapeutic tool. This trial will also determine the feasibility and applicability of a series of N-of-1 trials in a clinical discontinuation trial.

**Trial registration:**

ClinicalTrials.gov: NCT05051995, first registered September 20, 2021.

**Supplementary Information:**

The online version contains supplementary material available at 10.1186/s12888-023-05184-y.

## Background

Discontinuation of antidepressant medication has been associated with a wide range of physical and psychological symptoms. Physical complaints include flu-like symptoms, sleep disorders, gastro-intestinal symptoms, sensory disturbances, imbalance, and hyperarousal [1]. Psychological symptoms comprise emotional blunting, irritability, anxiety, poor concentration, and many more [2, 3]. The average incidence of discontinuation symptoms is 56% among patients with fully remitted depressive disorder [1]. Nearly half (46%) of those discontinuation symptoms are experienced as severe reactions [1]. Discontinuation symptoms are particularly common after discontinuation of the most widely prescribed antidepressants i.e. selective serotonin reuptake inhibitors (SSRIs) and serotonin-noradrenaline reuptake inhibitors (SNRIs) with incidence rates of up to 78% [2, 3]. Discontinuation symptoms typically have a rapid onset and usually subside within several weeks; yet, for some patients, discontinuation symptoms persist over several months [1, 2, 4].

The exact biochemical mechanisms underlying discontinuation symptoms remain largely unknown. Occurrence and severity are likely influenced by pharmacological and psychological factors. A shorter half-life, abrupt discontinuation, and higher maintenance dose are associated with a higher risk of discontinuation symptoms [5–8]. Longer antidepressant use, severity of original disease and individual factors (e.g. age, female gender) may also be relevant factors, though evidence is not unequivocal [9–11]. Antidepressant trials show strong nocebo effects (i.e. negative effects of an inert treatment caused by expectations and learning) with 38–100% of reported side-effects being accountable for by nocebo effects, suggesting the role of treatment expectations [12–15]. Similarly, qualitative studies reveal that negative expectations may evolve from a fear of relapse and negative pre-experiences with antidepressant discontinuation [16, 17]. Learning about negative discontinuation experiences of other users on social media might addtionally fuel negative expectations towards the own discontinuation process [18].

At the same time, placebo effects (i.e. positive effects of an inert treatment caused by positive expectations and learning) are common in antidepressant trials. Several meta-analyses show that the sole pharmacological effect of antidepressant medication is relatively small, and placebo treatments show similar benefits concerning reduction of depressive symptoms as antidepressant medication [19, 20]. However, harnessing these placebo effects by providing patients with deceptive placebos is ethically questionable. Recent evidence suggests deception may not be necessary, as open-label placebos (OLP) show to be beneficial for a wide range of disorders [21]. A recent meta-analysis documented a large effect size (SMD = 0.72, 95% CI 0.39–1.05, *p* < 0.001) of OLP compared to no treatment for various conditions, such as chronic back pain, cancer-related fatigue and major depression [22]. OLP treatment can reduce depressed mood, irritablility, fatigue, symptoms of a common cold, hot flushes, anxiety and pain [23–30]. These symptoms also commonly occur during or after discontinuation of antidepressant medication. Therefore, OLP treatment may alleviate discontinuation symptoms and possibly facilitate the discontinuation process.

Recruitment of patients with stable remitted depression who are willing to discontinue their antidepressant medication may be challenging and requires a cost-intensive design to examine the efficacy of OLP treatment in reducing antidepressant discontinuation symptoms. It is, therefore, difficult to achieve the required sample size in a randomized controlled trial (RCT). Yet, N-of-1 trials offer a pragmatic and high-integrity methodology in small patient groups [31]. N-of-1 trials are randomized, controlled, multiple crossover trials that investigate the effect of a certain treatment compared to a control condition in a single patient [32, 33]. As such, patients serve as their own control and therefore N-of-1 trials appear to be valuable in providing stringent evidence, particularly when estimating individual treatment effects [32, 34]. Aggregating a series of N-of-1 trials, e.g. by using Bayesian multi-level models, yields estimates of treatment effects at population-level [35–37]. Aggregated N-of-1 trials are particularly suitable for small patient populations, as they require a reduced sample size to estimate the overall treatment effect compared to an RCT due to the multiple crossover design [38, 39]. For this purpose, the current study applies a series of N-of-1 trials to examine the efficacy of OLP treatment in reducing antidepressant discontinuation symptoms. The results will provide first evidence concerning whether OLP treatment is an effective therapeutic tool in supporting the discontinuation of antidepressant medication.

### Objectives

The primary aim of this study is to investigate the efficacy of OLP treatment in reducing antidepressant discontinuation symptoms.

The following objectives will be investigated:

First, we will investigate individual- and population-level effect estimates of the efficacy of OLP relative to no treatment in reducing antidepressant discontinuation symptoms (primary outcome), and in improving dysfunctional treatment expectations, depressed mood and anhedonia (secondary outcomes) based on a series of N-of-1 trials. Second, we will explore factors that modify the efficacy of OLP treatment, i.e. age, female gender, prior negative experiences with discontinuation, discontinuation symptom load over the discontinuation period, higher maintenance dose and longer duration of antidepressant use.

## Methods

### Study design

**Fig. 1:**
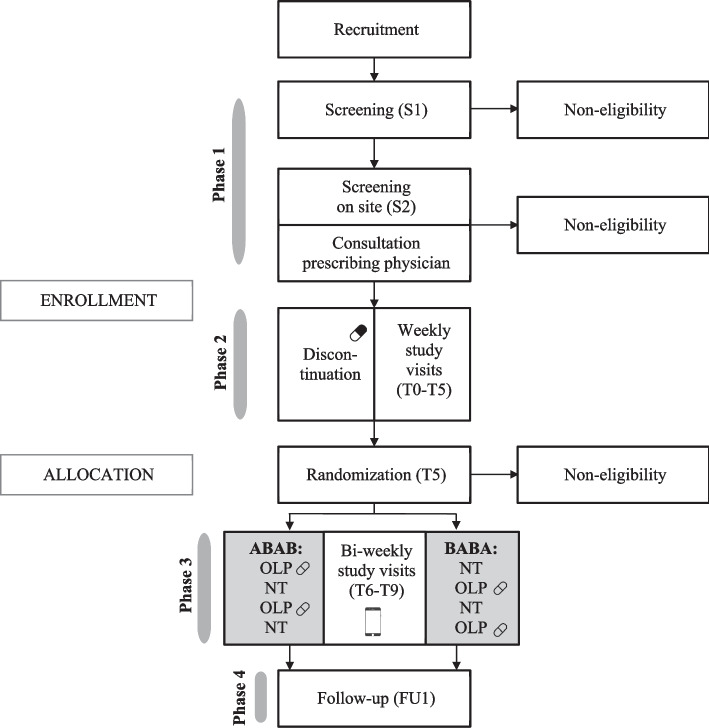
Participant flow and study design. S1 = telephone screening (interview: sociodemographic and medical characteristics); S2 = on-site screening (clinical interviews: sociodemographic and medical characteristics, Adverse Events (AE), Montgomery-Åsberg Depression Rating Scale (MADRS), Structured Clinical Interview for DSM-V - Clinician Version (SCID-V-CV); questionnaire: Beck Depression Inventory-II (BDI-II)); T = timepoint study visit; T0 = baseline study visit (week 0) at the start of the discontinuation phase (clinical interviews: MADRS, adherence, AE, single safety questions; online questionnaires: Generic Rating Scale for Previous Treatment Experiences (GEEE_PRE_), Generic Assessment of Side-Effects (GASE), Discontinuation Emergent Signs and Symptoms Scale (DESS_PAST_), BDI-II, Short Warwick-Edinburgh Mental Wellbeing Scale (SWEMWBS), State-Trait Anxiety-Depression Inventory (STADI), Treatment Expectation Questionnaire (TEX-Q), Generic Rating Scale for Treatment Expectations (GEEE_EXP_), Perceived Stress Scale (PSS), sociodemographic characteristics); T1-T5 = weekly study visits (week 1-5) during the discontinuation phase (clinical interviews: MADRS, adherence, AE, single safety questions; online questionnaires: DESS, BDI-II, SWEMWBS, Generic Rating Scale for Treatment Effects (GEEE_ACT_), TEX-Q, GEEE_EXP_; blood collection (T1); randomization to experimental groups (T5)); T6-T9 = bi-weekly study visits (week 7, 9, 11, 13) during experimental phase (clinical interviews: MADRS, adherence, AE, single safety questions; online questionnaires: DESS, BDI-II, SWEMWBS, GEEE_ACT_, TEX-Q, GEEE_EXP_, GASE (T9); blood collection (T9); implementation of N-of-1 trials (week 6-13) including treatment interventions (A: Open-Label Placebo (OLP); B: No Treatment (NT)), and daily ambulatory assessments: GEEE_ACT_, GEEE_EXP_, Patient-Health-Questionnaire-2 (PHQ-2)); FU1 = Follow-up (week 26; telephone interview: MADRS, adherence, AE, single safety questions; online questionnaires: DESS, BDI-II, SWEMWBS, GEEE_ACT_, GEEE_EXP_); A total of 11 study visits (S2-FU1) per participant including 6 hours of clinical interviews, and 6 hours of questionnaires are planned

To assess the efficacy of OLP compared to no treatment in reducing discontinuation symptoms, a series of randomized, single-blinded, multicenter N-of-1 trials will be conducted. Each trial consists of four study phases: (1) screening and eligibility; (2) a five-week guided, gradual discontinuation phase in line with German guideline recommendations, including a one-week run-in phase; (3) eight-week N-of-1 experimental phase, and (4) a follow-up assessment six months after baseline (Fig. 1). After successful discontinuation, 20 patients with moderate to severe discontinuation symptoms will be included in the N-of-1 trials and randomly allocated to two groups, differing in treatment order of OLP and no treatment. Results of the individual N-of-1 trials will be combined to produce population estimates for the efficacy of OLP treatment using a hierarchical Bayesian model [40]. This study is part of a collaborative research center (CRC; TRR 289 Treatment Expectation: treatment-expectation.de/en/).

### Interventions

During the eight-week experimental phase, the series of N-of-1 trials including the treatment intervention will be implemented. Each N-of-1 trial consists of two-week periods of either OLP or no treatment in an alternating order. Patients will be randomized to either OLP or no treatment as their starting intervention and alternate between these two conditions during the experimental phase (i.e. ABAB/BABA, where ‘A’ represents OLP and ‘B’ no treatment). At the start of the OLP treatment period, patients will receive placebo tablets (1ml gelantine capsules with filler material (99,5% mannitol, 0,5% silicon dioxide), manufactured by the pharmacy of each study site) enclosed in tabular film, and are instructed to take one placebo tablet two times per day for the following two weeks. Patients will be provided with an OLP rationale in accordance with Kaptchuk et al. (2010) with a standardized manual applied by trained study staff (approx. 15 min) [21]. The rationale comprises four discussion points: (1) the placebo effect is strong; (2) the body can automatically react to taking placebo pills; (3) a positive attitude helps but is not necessary; (4) a regular intake is necessary [21]. Based on evidence for a positive influence of habits on medication adherence, a fifth discussion point is added: (5) combine the OLP intake with a habit [41]. Patients will additionally receive a leaflet summarising the five discussion points. During the period of no treatment, patients will receive no further instruction.

### Randomization and blinding

A block-randomization will be applied for the two study sites. Patients will be randomized in a 1:1 ratio to either the treatment order ‘ABAB’ (OLP; no treatment; OLP; no treatment) or ‘BABA’ (no treatment; OLP; no treatment; OLP). Allocation sequence will be based on computer-generated random numbers in R-Studio with the package *blockrand* [42]. Patients and study staff responsible for the randomization procedure will not be blinded to treatment allocation. Unblinded study staff will inform the patients about treatment allocation and provide specific instructions after each study visit. Study staff involved in further assessements (i.e. clinical interviews) will be blinded to treatment allocation, and patients will be asked not to inform the assessor whether they currently receive OLP or no treatment to ensure unbiased assessment.

### Participants

Patients will be recruited via leaflets, regional advertisement, (social) media, emails to university students in Marburg, via cooperating general practioners, and psychiatric outpatient clinics. Individuals interested in study participation can approach the study team via email, phone, or the study’s web page. We will recruit patients who have experienced one or multiple depressive episodes, report antidepressant use, and attained stable remission.

### Inclusion criteria

- Adult patients (≥ 18) with fully remitted major depressive disorder (MDD), single or recurrent, confirmed by the Structured Clinical Interview for DSM-V - Clinician Version (SCID-V-CV) [43];
- Antidepressant use (citalopram (20-40 mg), duloxetine (60-100 mg), escitalopram (10-20 mg), paroxetine (20-40 mg), sertraline (50-150 mg), venlafaxine (75-150 mg) or mirtazapine (30-45 mg)) with a constant dosage for four weeks;
- Discontinuation wish by participant, acknowledged by prescribing physician;
- Fulfilment of the S3 German national guideline recommendations to discontinue antidepressant medication: (a) response to antidepressant medication; (b) symptom remission ≥ 4 months (first episode) or ≥ 2 years (≥ 2 episodes with significant functional impairment) [44];
- Informed consent;
- At least moderate discontinuation symptoms after antidepressant discontinuation assessed by the Generic Rating Scale for Treatment Effects (GEEE_ACT_ score ≥ 4/10 during past week) [45].

### Exclusion criteria

- Current moderate or severe psychopathological symptoms or psychosocial impairments;
- Acute or chronic somatic illness which might interfere with depressive disorder, antidepressant use or proposed study;
- Acute suicidality, psychotic symptoms, substance abuse or addiction, current mania or hypomania confirmed by SCID-V-CV or other psychopathology which might interfere with depressive disorder, antidepressant use or proposed study;
- Any history of bipolar disorder or psychosis confirmed by SCID-V-CV;
- Severe stressful life events within six months prior to study participation;
- Current pregnancy;
- Insufficient German language proficiency.

### Sample size

Sample size calculations are based on the main analysis concerning the efficacy of OLP treatment compared to no treatment in reducing discontinuation symptoms assessed on a 11-point numerical rating scale (GEEE_act_) [45]. For the analysis resulting in population-level estimates of the treatment effect, sample size calculations were performed using the Shiny-App (https://jiabeiyang.shinyapps.io/SampleSizeNof1/) which implements a linear mixed model specifically designed for sample size calculations in a series of N-of-1 trials [46]. We assume a fixed intercept and random slope model for alternating sequences (i.e. ABAB/BABA). We used the daily GEEE_ACT_ scores for a fixed number of patients (*N* = 20) and a fixed number of measurements per patient (112 measurements) with a homogeneous residual standard error of 1.5, variance of random slope of 0.75, and an autocorrelation for repeated measures (first-order autocorrelation (AR-1) structure) of 0.7. The results of the sample size calculation suggest that a sample size of 18 participants yields a power of 93% for identifying a mean difference between OLP treatment and no treatment when the mean difference is 0.8 points (on the 11 point GEEE_ACT_ rating scale) which we consider as minimally clinically important difference (at a significance level of 5%). Considering possible dropout, we will recruit 20 patients into the study. This study design can be expected to yield naïve (i.e. non-pooled) estimates of the individual treatment effects with a standard error of about 0.3, hence allowing also meaningful inference on the individual level.

### Procedure

Assessments will be conducted at the University Medical Center Hamburg-Eppendorf, Institute of Systems Neuroscience (Hamburg, Germany) and the Philipps-University Marburg, Department of Psychiatry and Psychotherapy (Marburg, Germany), and standardized across sites according to pre-defined standard operating procedures (SOPs). Figure 1 shows the study design and participant flow through the trial.

### Phase 1: screening and eligibility (S1-S2)

First, a telephone screening (S1; approx. 10–15 min) will be conducted to assess sociodemographic and medical characteristics central for eligibility. Potentially eligible patients will then be invited for a thorough on-site eligibility screening (S2). During S2, patients will be informed about study procedures. Clinical interviews and questionnaires will be applied to assess sociodemographic and medical characteristics, current depressive symptoms, psychopathology, and adverse events (approx. 90 min). Finally, the prescribing physician will be consulted to confirm medical characteristics and approve initation of antidepressant discontinuation. All eligible participant provide written informed consent and will then be enrolled for study participation.

### Phase 2: discontinuation (T0-T5)

After enrollment, the discontinuation process will be initiated with concurrent weekly study visits. Each study visit will include clinical interviews, online questionnaires, patient-practitioner interaction, and supply of the study medication (approx. 60 min each). Discontinuation schedules will be individualized and tailored for a guided, gradual discontinuation process. Each discontinuation process starts with a run-in phase. Patients receive their maintenance dose, though newly encapsuled, for one week to control for effects caused by the different appearance or haptic of the study medication. Subsequently, the dosage will be gradually reduced over a four-week period with five dose reduction steps. Reduction steps will be greater in the beginning followed by smaller dose reductions as dosages approach zero, in line with recommendation for hyperbolic dose reductions [47]. Discontinuation schedules are based on the S3 German national guideline recommendations effective at the time of study start, suggesting a four-week gradual reduction [44]. Patients will receive study medication weekly at study visits. Following successful discontinuation (T5) the eligibility for the N-of-1 trials will be evaluated, based on the GEEE_ACT_ ratings assessing discontinuation symptoms [45].

### Phase 3: N-of-1 trials (T6-T9)

During the third study phase, bi-weekly study visits are scheduled (T6-T9) that include clinical interviews and questionnaires (approx. 60 min). If patients are not eligible for the N-of-1 trials, assessments serve as a clinical observation phase. If patients are eligible for the N-of-1 trials, they will be first informed about the N-of-1 trial procedure by unblinded study staff. Patients will then receive a leafleat with information on their group allocation and about participation in ambulatory assessment. Ambulatory assessments will be conducted using the ‘StudyU’ application, and include measurements twice daily, i.e. in the morning (8.00-10.00am) and evening (6.00-8.00pm), over eight weeks [48]. The ‘StudyU’ application will contain information about current treatment allocation, study progress and notifications to remind patients about assessments. At the beginning of the first OLP treatment, patients will receive placebo tablets with verbal and written OLP rationale (approx. 15 min). Additionally, the rationale will be refreshed at the start of the second OLP period.

### Phase 4: follow-up (FU1)

Six months after study start, patients will be contacted for a short clinical assessment (approx. 45 min). Clinical interviews will be administered online or via telephone, and questionnaires will be assessed online. Afterwards patients will receive compensation based on time expenditure for study participation of up to 90 Euro.

### Outcomes

Outcomes will be assessed via clinical interviews at the study centers, via online questionnaires using Lime Survey (Lime Survey, Hamburg, Germany), and ambulatory assessments using the ‘StudyU’ application [48]. An overview of all outcome assessments in accordance with the SPIRIT guidelines is depicted in Table 1 [49].

**Table 1:**
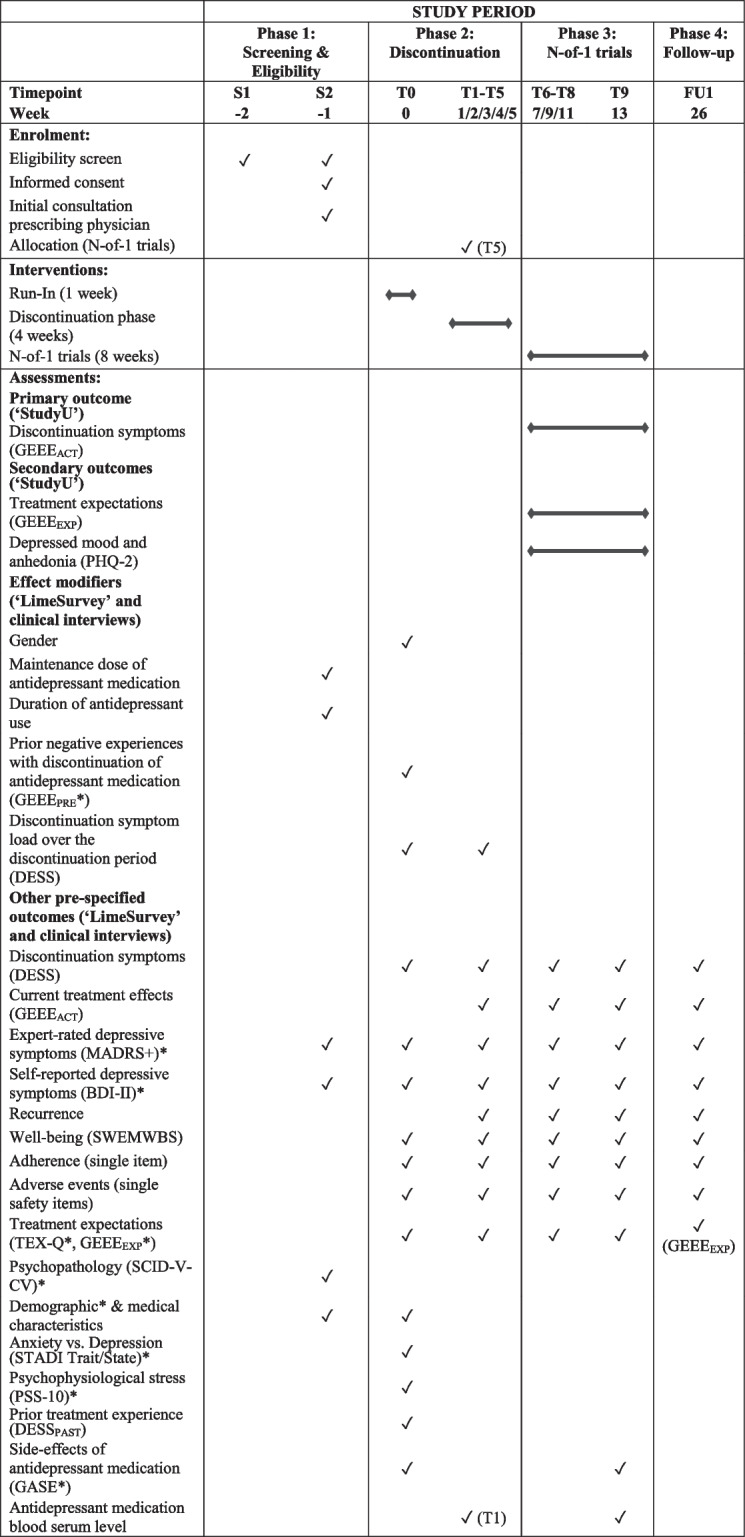
Schedule of enrollment, intervention, and assessments according to SPIRIT-PRO

*S* Screening, *T *Timepoint (study visit), *FU *Follow-Up, *GEEE*_ACT_ Generic Rating Scale for Treatment Effects, *GEEE*_EXP_ Generic Rating Scale for Treatment Expectations, *PHQ-2 *Patient-Health-Questionnaire-2, *GEEE*_PRE_ Generic Rating Scale for Previous Treatment Experiences, *DESS *Discontinuation Emergent Signs and Symptoms Scale; *MADRS* = Montgomery-Åsberg Depression Rating Scale; *BDI-II* = Beck Depression Inventory-II; *SWEMWBS* = Short Warwick-Edinburgh Mental Well-Being Scale; *TEX-Q* = Treatment Expectation Questionnaire; *SCID-V-CV* = Structured Clinical Interview for DSM-V - Clinician Version;* STADI* = State-Trait Anxiety-Depression Inventory; *PSS-10 *= Perceived Stress Scale-10; *DESS*_PAST_ = Discontinuation Emergent Signs and Symptoms Scale (previous experiences); *GASE *= Generic Assessment of Side-Effects

### Primary outcome measure

*Discontinuation symptoms* will be assessed twice daily during the eight-week (T5-T9) N-of-1 trials (112 assessments per person in total) via the ‘StudyU’ application using a modified, single item of the *Generic Rating Scale for Treatment Effects* (GEEE_ACT_). Patients will be asked to rate how many complaints/side-effects caused by the discontinuation of antidepressant medication they experienced since the last assessment. Intensity will be rated on a 11-point numeric rating scale (NRS) ranging from 0 to 10 (0 ‘*no side-effects’* − 10 ‘*greatest side-effects imaginable*’) [45]. Higher scores indicate greater discontinuation symptoms.

### Secondary outcome measures

*Treatment expectations* will be assessed twice daily via the ‘StudyU’ application during the N-of-1 trials (T5-T9) using a modified, single item of the *Generic Rating Scale for Treatment Expectations* (GEEE_EXP_). Patients will be asked to rate how many complaints/side-effects caused by the discontinuation of antidepressant medication they expect until the next assessment. Intensity will be rated on a 11-point NRS ranging from 0 to 10 (0 ‘*no side-effects*’ − 10 ‘*greatest side-effects imaginable*’) [45]. *Depressed mood and anhedonia* will be examined twice daily via the ‘StudyU’ application during the N-of-1 trials (T5-T9) using the *Patient-Healthcare-Questionnaire-2* (PHQ-2). The PHQ-2 consists of 2 items scored on a 4-point Likert-scale with a range of 0–3 (0 ‘*not at all*’ − 3 ‘*nearly all the time*’) [50]. Thus, a total score range of 0–6 with higher scores suggestive of a higher degree of depressive symptomatology.

### Possible effect modifiers

Age, female gender, prior negative experiences with discontinuation, discontinuation symptom load over the discontinuation period, duration of antidepressant use, and maintenance dose will be explored as possible effect modifiers. *Age* (years) and *gender* (female, male, non-binary) will be assessed via self-report as single items. *Prior negative experiences with discontinuation* will be measured with an adjusted version of the *Generic Rating Scale for Previous Treatment Experiences* (GEEE_PRE_) at T0 [45]. Negative experiences of the latest discontinuation attempt will be rated on an 11-point NRS (0 ‘*no worsening*’ − 10 ‘*greatest worsening imaginable*’). Higher scores indicate more negative prior experience with discontinuation. *Discontinuation symptom load over the discontinuation period* will be based on the average total score of the *Discontinuation Related Signs and Symptoms Scale* (DESS) during the discontinuation phase (T1-T5) [51]. The DESS is a self-report measure comprising 43 discontinuation symptoms rated on a 4-point scale (0 ‘*not present*’ − 3 ‘*severe*’) with a total range of 0-129. Higher scores indicate more severe discontinuation symptoms. *Duration of antidepressant use* (years) and *maintenance dose* will be assessed at study start (S2) via self-report, with the maintenance dose additionally confirmed by the prescribing physician.

### Other pre-specified outcome measures

We will include additional assessments as part of the collaborative research center CRC/TRR 289 ‘treatment expectation’, contributing to large-scale pooled analyses [52]. These include sociodemographic characteristics, treatment experiences (GEEE_ACT_; [45]), treatment expectations (TEX-Q, GEEE_EXP_; [45, 53]), state/trait anxiety and depression (STADI; [54]), psychopathological stress (PSS-10; [55]); psychopathology (SCID-V-CV; [43]), and side-effects of antidepressant use (GASE; [56]). Further assessments include discontinuation symptoms over the course of the study (DESS; [51]), and mental well-being (SWEMWBS; [57]).

### Safety-endpoints

*Depressive symptomatology* will be assessed at screening and study visits (S2-FU1) via expert-rating of the *Montgomery-Åsberg Depression Scale* (MADRS), which is a clinical interview comprising 10 items with intensity ratings from 0 to 6 with specific anchors [58]. Total scores range between 0 and 60. Higher scores indicate more severe depressive symptomatology. The *Structured Interview Guide for the MADRS* (SIGMA) will be used to standardize the assessements [59]. *Depressive symptoms* will additionally be assessed at the screening and study visits (S2-FU1) via self-report with the *Beck Depression Inventory-II* (BDI-II) [60]. The BDI-II consists of 21 items each rated on 4 response options. Total scores range between 0 and 63. Higher scores indicate more severe depressive symptomatology. *Recurrence* will be assessed during the course of the study (T1-FU1) based on the total MADRS score > 21 or total BDI-II score > 19 at two consecutive study visits, and confirmed by the SCID-V-CV section A [43]. *Adverse events* (AE) will be assessed at study visits (S2-FU1) by a single question (e.g. *‘Did you experience any adverse events since our last study visit?’*). AEs will be classified by trained study staff according to the *Common Terminology Criteria for Adverse Events* (CTCAE) [61]: grade 1 ‘*mild*’, grade 2 ‘*moderate*’, grade 3 ‘*severe*’, grade 4 ‘*life-threatening*’, grade 5 ‘*death*’. Similarly, the attribution of the AE to study participation will be assigned in accordance with the *World Health Organization* [62].

*Adherence* will be assessed at study visits (T0-FU1) by a single question, i.e. during the discontinuation phase ‘*On how many days since the last study visit did you take the medication*?’ which will be adjusted for the placebo intake during the N-of-1 trials. During follow-up a single question about antidepressant use will assess adherence, i.e. ‘*Did you abstain from antidepressant medication since the last study visit?*’. Blood samples will be collected at T1 and T9 to assess the antidepressant medication blood serum level. The blood collection will be performed either in the morning or in the evening before antidepressant intake (approx. 24 h after the last antidepressant intake) by trained study staff. Blood analysis will be accomplished by the Department of Legal Medicine, University Medical Center Hamburg-Eppendorf. The analysis will be based on the Liquid Chromatography-Mass Spectrometry (LC-MS/MS) [63]. Therefore, the multiple reaction monitoring (MRM) mode guarantees a high specificity and sensitivity. The blood serum level will serve as additional indicator for adherence.

### Data management

Data collection and handling will be in accordance with the European General Data Protection Regulation (GDPR). Trial specific documents will be stored securely with access restricted to nominated research staff. Personal patient data will be stored locally at each site in accordance with governmental regulations. Only principal investigator and nominated research staff will have access to the data. Data of the digital questionnaires (Lime Survey), smartphone assessments (‘StudyU’) and clinical assessments (paper form) will be pseudonymised throughout the whole study duration. Data will be pseudonymized with the ALIIAS software comprising a two-factor authentication, deterministic pseudonymization technique [64]. Data from Lime Survey will be stored on a server provided by the University of Duisburg-Essen, Essen, Germany and will be securely stored in a cloud server with limited access for nominated research staff. Data from the ‘StudyU’ application will be encoded via individual invite codes that are not linked to any personal data. The ‘StudyU’ application will not collect identifiable information. The anonymized data from the ‘StudyU’ application will be saved directly on a safe backend hosted by the Hasso-Plattner-Institute, Potsdam, Germany and will be stored locally at each site [48]. Patients are requested to download the ‘StudyU’ application to confidentially answer the daily questionnaires. For the sake of participant retention, notifications will serve as reminder. Patients will be asked to agree with the terms of use of the ‘StudyU’ application consisting information about data storage and publication [48]. Patients will be informed that the app can be deleted after study completion.

### Statistical analysis

A detailed statistical analysis plan (SAP) will be established and published on ClinicalTrials.gov prior to analysis. The primary analysis will obtain individual- and population-level estimates of the efficacy of OLP treatment in reducing discontinuation symptoms based on the series of N-of-1 trials. This analysis will be based on the intention-to-treat population.

For the individual N-of-1 trials, Bayesian models will be applied to compare the effect of OLP to no treatment in reducing discontinuation symptoms (primary outcome). These obtained estimates of individual-level effects can be viewed as naïve estimates as they do not incorporate information from the other participants. These models will include a first-order autoregressive (AR1) error structure to consider that measurements at adjacent times show a higher correlation than measurements far apart in time given the longitudinal nature of the data in our study. Non-informative priors will be selected for all parameters (as there was no prior information available before the study that allowed a reasonable effect estimate of OLP in reducing discontinuation symptoms). For each individual, separate analyses will be conducted, comparing the average response to each treatment. Based on this model we will obtain estimates of the posterior distribution of the average OLP treatment effect at individual-level. Secondary outcomes will be analyzed similarly.

For the aggregation of the N-of-1 trials, a Bayesian multi-level model will be applied to assess the efficacy of OLP relative to no treatment in reducing discontinuation symptoms (primary outcome) at population-level. We will utilize multi-level mixed models to estimate the posterior distribution of the population-level average treatment effect and the within- and between-patient variance. Additionally, we will obtain a pooled estimate of the individual-level patient’s treatment effect. For the aggregated analysis, non-informative priors will be selected for all parameters, as there was no prior information available before the study that allowed a reasonable effect estimate of OLP treatment in reducing discontinuation symptoms. The dependence of responses over time will be modeled with an AR1 error structure. Similarly, to the individual-level model described above, we will obtain estimates of the posterior distribution of the average OLP treatment effect on the population-level in reducing discontinuation symptoms. Extensions to the above model will be applied to account for time trends. Further, we will assess between-patient covariates such as age, female gender, prior negative pre-experiences with discontinuation, discontinuation symptom load over the discontinuation period, higher maintenance dose, and duration of antidepressant use to determine if the given outcome is related to such variables. From the aggregated Bayesian analysis we derive the posterior distribution of the mean difference between the outcomes of OLP and no treatment. Secondary outcomes will be modelled in the same manner. Statistical analyses will be performed in JAGS running from R, using the Markov Chain Monte Carlo (MCMC) method to obtain empirical samples from the joint posterior distribution of the parameters.

### Monitoring and safety

All safety-relevant measures will be administered and evaluated at each study visit (T0-FU1) by trained study staff. Patients can leave the study at any time for any reasons. Treatment will be terminated in case of pregnancy, withdrawal of informed consent, or objections by study staff based on health risks or insufficient compliance. Health risks may include occurrence of a treatment-related serious AE, recurrence of major depression, acute suicidality, and severe discontinuation symptoms. In case of termination of study participation, further treatment options will be discussed and patients will nevertheless be invited to complete all measurement points. If patients are not able to attend a study visit due to illness or otherwise, medication will be delivered by study staff. If illness interferes with study participation, adaptions to the discontinuation plans with an extension of up to seven weeks in total are possible. Therefore, the dose reduction will be interrupted for the extension period. Patients will be requested to return all unused medication including tabular films to the clinic. A pre-defined plan of safety procedures will be administered throughout the study and includes three safety stages: stage 1 ‘*mild*’, stage 2 ‘*moderate*’, stage 3 ‘*severe*’. In case of mild safety concerns (stage 1: mild AE; severe distress relating to study treatment; suicidal ideation; abnormal clinical impression), a three-week monitoring procedure with additional weekly study visits will be initiated. In case of moderate safety concerns (stage 2: moderate AE; moderate depressive symptoms; suicidal thoughts; abnormal clinical impression), a six-week intensive monitoring will be initiated with additional weekly study visits. In case of severe safety concerns (stage 3: serious AE; severe depressive symptoms; acute suicidality; abnormal clinical impression), study treatment will be terminated, and immediate psychiatric treatment will be initiated. For further details regarding safety procedures, see Meißner et al. 2023 [65]. After completion of phase 3, prescribing physicians of the patients will be informed about successful discontinuation of antidepressant medication to ensure long-term patient well-being.

#### Data and safety monitoring board

An independent data and safety monitoring board (DSMB) will advise and review the proposed study. Serious AEs, severe depressive symptomatology, acute suicidality or other relevant medical events will be reported immediately (within 48 h) to the DSMB. The DSMB will receive bi-annual reports to analyze patients safety and make recommendations regarding the continuation, modification, or termination of the study.

## Discussion

This trial will provide first evidence of the applicability of OLP as treatment in reducing antidepressant discontinuation symptoms. To investigate efficacy, a series of N-of-1 trials will be implemented. Within each N-of-1 trial, patients who successfully discontinued antidepressant use will undergo two cycles of OLP and no treatment in a random order for a course of eight weeks. Patients will be asked to rate discontinuation symptoms twice daily via a smartphone application. Bayesian analyses will be applied to estimate the treatment effect of OLP treatment in reducing antidepressant discontinuation symptoms at individual- and population-level. Additional analyses will reveal the effect of OLP treatment on treatment expectations and depressive symptoms. Effect modifiers such as age, gender, prior negative experiences, discontinuation symptom load over the discontinuation period, maintenance dose and duration of antidepressant use will be investigated. Our approach will allow to identify the treatment effect of OLP consistently during the discontinuation process at individual- and population-level.

One of the main strengths of the present study is the opportunity to obtain evidence for a treatment in a clinical discontinuation trial, at individual- and group-level simultaneously using Bayesian analyses of N-of-1 trials. Further, the aggregation of the N-of-1 trials yields an estimation of the overall treatment effect with a smaller sample size required than in traditional RCTs, which is particularly advantageous in cost-intensive designs in a population that is challenging to recruit [66]. Another strength is the standardized, controlled discontinuation process combined with extensive assessments. All patients will gradually discontinue their antidepressant medication over a course of four weeks according to pre-defined discontinuation schedules with study medication that is standardized in terms of appearance and packaging. During and after the discontinuation process a thorough assessment of clinical features, and an extensive psychometric battery will be applied. This allows for a consistent investigation of factors contributing to the individual experiences during antidepressant discontinuation.

It should be noted that this study protocol is not without limitations. A difficulty is the risk of patients dropping out, increased by the expected impact of the discontinuation process. The regular study visits and a highly elaborated safety plan will encourage adherence to protocol. Additionally, analyses will be based on an intention-to-treat method considering the different discontinuation experiences. Furthermore, the fluctuations in the discontinuation symptoms contribute to difficulties in the analysis of the N-of-1 trials. The randomization with different treatment orders aims to balance for this effect, though time trends need to be considered in the analyses.

Discontinuation symptoms pose a major clinical challenge to successful antidepressant discontinuation. Therapeutic and medical tools to support patients and caregivers during the discontinuation process are needed. OLP are effective in reducing various physical and psychological symptoms that are also associated with antidepressant discontinuation [22]. Our trial will provide insight into whether harnessing the placebo effect by using OLP treatment to alleviate discontinuation symptoms can aid successful antidepressant discontinuation.

### Trial status

The study is currently recruiting.

### Supplementary Information

**Additional file 1.**

**Additional file 2.**

## Abbreviations

AE: Adverse event
BDI-II: Beck Depression Inventory-II
CRC: Collaborative Research Center
CTCAE: Common Terminology Criteria for Adverse Events
DESS: Discontinuation Emergent Signs and Symptoms Scale
DFG: German Research Foundation
DSMB: Data and Safety Monitoring Board
FU: Follow-Up
GASE: Generic Assessment of Side-Effects
GDPR: European General Data Protection Regulation
GEEE: Generic Rating Scale for Previous Treatment Experiences, Treatment Expectations, and Treatment Effects
LC-MS/MS: Liquid Chromatography-Mass Spectrometry
MADRS: Montgomery-Åsperg Depression Rating Scale
MRM: Multiple Reaction Monitoring
NRS: Numeric Rating Scale
OLP: Open-Label Placebo
PHQ-2: Patient-Healthcare-Questionnaire-2
PSS-10: Perceived Stress Scale 10
RCT: Randomized Controlled Trial
S: Screening
SAP: Statistical Analysis Plan
SCID-V-CV: Structured Clinical Interview for DSM-V – Clinician Version
SIGMA: Structured Interview Guide for the MADRS
SOP: Standard Operating Procedure
SNRI: Serotonin-Noradrenaline Reuptake Inhibitors
SSRI: Selective Serotonin Reuptake Inhibitors
STADI: State-Trait Anxiety-Depression-Scale
SWEMWBS: Short Warwick-Edinburgh Mental Well-Being Scale
T: Timepoint (study visit)
TEX-Q: Treatment Expectation Questionnaire
