# Supplementary material for: Study protocol: combined N-of-1 trials to assess open-label placebo treatment for antidepressant discontinuation symptoms [FAB-study]
Source: BMC Psychiatry. 2023 Oct 13;23:749. doi: 10.1186/s12888-023-05184-y (PMC10576328; doi:10.1186/s12888-023-05184-y)
Supplement: Supplementary file 2 — Additional file 2. [file 12888_2023_5184_MOESM2_ESM.docx]

Summary of Registration according to WHO Trial Registration Data Set

|  | |  |
| --- | --- | --- |
| **Data category** | **Information** | |
| Primary registry and trial identifying number | ClinicalTrials.gov  NCT05051995 | |
| Date of registration in primary registry | September 20, 2021 | |
| Secondary identifying numbers | 0721-20  PV7151 (Ethics Approval Number)  CRC 289 Project A15 (Other Grant/Funding Number)  U1111-1274-2336 (Registry Identifier) | |
| Source(s) of monetary or material support | This research is funded by the Deutsche Forschungsgemeinschaft (DFG, German Research Foundation): TRR 289 Treatment Expectation - Project Number 422744262. | |
| Primary sponsor | Universitätsklinikum Hamburg-Eppendorf (University Medical Centre Hamburg-Eppendorf; please contact [y.nestoriuc@hsu-hh.de](mailto:y.nestoriuc@hsu-hh.de) (Principal investigator) for any queries) | |
| Secondary sponsor(s) | n.a. | |
| Contact for public queries | YN, [y.nestoriuc@hsu-hh.de](mailto:y.nestoriuc@hsu-hh.de) (Principal investigator) | |
| Contact for scientific queries | YN, [y.nestoriuc@hsu-hh.de](mailto:y.nestoriuc@hsu-hh.de) (Principal investigator) | |
| Public title | Combined N-of-1 Trials to Assess Open-Label Placebo Treatment for Antidepressant Discontinuation Symptoms | |
| Scientific title | Combined N-of-1 Trials to Assess Open-Label Placebo Treatment for Antidepressant Discontinuation Symptoms | |
| Countries of recruitment | Germany | |
| Health condition(s) or problem(s) studied | Depressive Symptoms  Expectations  Antidepressants | |
| Intervention(s) | Drug: Open-label placebo treatment  Patients receive placebo tablets with the instruction to take 2 tablets daily for 2 weeks. Prior to the open-label placebo administration, an explanation why placebos without concealment might be effective is offered.  Other: No-treatment  During the no-treatment phase, participants receive no treatment for 2 weeks | |
| Key inclusion and exclusion criteria | Key inclusion criteria   - Adult patients (≥18 years) with fully remitted MDD, single or recurrent; - Antidepressant use (SSRI/SNRI or NaSSA); - Discontinuation wish by patient, acknowledged by prescribing physician; - Fulfilment of S3 German guideline recommendations to discontinue antidepressant medication. - Informed consent.   Key exclusion criteria   - Moderate or severe psychopathological symptoms; - Acute or chronic somatic illness; - Acute suicidality; - History of bipolar disorder or psychosis; - Insufficient German language proficiency; | |
| Study type | Interventional  Allocation: Randomized Intervention Model: Crossover Assignment  Masking: Single (Outcomes Assessor)  Primary Purpose: Supportive Care | |
| Date of first enrolment | 05.04.2022 | |
| Target sample size | 20 | |
| Recruitment status | Recruiting | |
| Primary outcome(s) | Antidepressant discontinuation symptoms during the experimental phase (8 weeks) assessed twice daily (112 measurements per person) | |
| Key secondary outcomes | Secondary outcomes will include: symptom expectations during the experimental phase; depressed mood and anhedonia during the experimental phase | |
| Version protocol | Version 1: September 12^th^, 2019 (as submitted to ethics committee)  Version 2: October 29^th^, 2019 (resubmitted with minor changes for fulfilment of requirements relating to initial submission)  Version 3: March 30^th^, 2021 (1^st^ amendment to add a pilot study and change public study tile)  Version 4: July 15^th^, 2021 (2^nd^ amendment to add optional rsfMRI measurement)  Version 5: May 12^th^. 2023 (3^rd^ amendment to add changes following piloting the study (e.g., primary and secondary outcomes, safety endpoints, data and safety monitoring, statistical analyses) and resubmission with all prior changes tracked and summarized) | |
